# Editor’s Book Table

**Published:** 1871-04

**Authors:** 


### Editor's Book Table.

[NOTE.—All works reviewed in the columns of the CHICAGO MEDICAL JOURNAL may be found in the extensive stock of W. B. KEEN & COOKE, whose catalogue of Medical Books will be sent to any address upon request.]

*General Surgical Pathology and Therapeutics, in fifty lectures. A Text Book for Students and Physicians.* By Dr. THEODOR BILLROTH, Professor of Surgery in Vienna. Translated from the fourth German edition, with the special permission of the author, by Charles E. Hackley, A.M., M.D., Surgeon to the New York Eye and Ear Infirmary, Physician to the New York Hospital, Fellow of the New York Academy of Medicine, etc., etc. New York: D. Appleton and Company, 90, 92, and 94 Grand street. 1871.

The translator informs us in his preface that: "Most of the views found in these lectures have been floating through the journals for several years past; but, so far as the translator knows, they are not so fully presented in any book in the English language." This information was hardly necessary to those who will be likely to purchase the book, nor was it necessary as a reason for the work which he has so well performed. In fact, the circumstance of the floating condition of certain ideas is but a poor reason for giving them book form. It needs but the authority of Virchow, for instance, to set any idea permanently afloat in periodical literature; the idea itself may be wild, visionary, and even foggy, but the parentage will secure the propagation upon the pages of the journals and the tongues of teachers, without the accompanying criticism, which would be, at least, wholesome. A glaring fault of the profession, both in this country and England, for the past few years, has been to accept, unchallenged, the dicta of a few German authors.

We commenced the perusal of Dr. Billroth's lectures with great expectations, and although profit as well as interest has attended our steps, we confess to disappointment, the reasons of which will appear as this article progresses. The scope of the book differs from Paget's Surgical Pathology in that the author enters largely into symptomatology and therapeutics, thus rendering it acceptable to a larger class of readers.

On the subject of suppuration, Dr. Billroth coincides with Cohnheim in deriving his supply of pus corpuscles from the colorless blood corpuscles. In fact, the white-blood corpuscle plays the principal part in most pathological changes, whether of construction or destruction. Proliferation, which five years since was so popular, finds little favor with our author. Virchow proliferated his pus from the nuclei of connective tissue, as though the nuclei were possessed of *creative* power, and could multiply *ad infinitum*, independently of blastema or formative material. Billroth equally ignores formative material, except so far as it is represented by the white corpuscle or *wandering cells*. The following sentence will express his idea as pertinently as any single sentence can:

"If there be no longer any doubt that all young cells that infiltrate the inflamed tissue, and sometimes, as we shall hereafter see, escape from it in the shape of pus, are white-blood corpuscles, or, briefly, *wandering cells*, we have two questions to answer, namely, *Why* do so many cells wander into inflamed tissue, and how came these numbers of wandering cells in the blood; where do they originate?"

It is not our intention, in this notice, to combat or uphold the views of either Virchow or Billroth, but if suppuration is any process, it is, probably, a uniform process; it is not probable that suppuration is a radically varying process. If it is an "escape" of wandering cells in one instance, it is undoubtedly always the same process. But our author converts a blood clot into pus, also: "This blood clot may sometimes interfere with healing, as when, from its size or other causes, it decomposes or turns to pus." (p. 58.) He also converts tissue into pus: "The tissue surrounding the spot first diseased is gradually infiltrated with cells; and it also goes on to form fluid cellular tissue with the character of pus." (p. 368.) Suppuration is also, according to our author, a *healing* process. He says: "For instance, you unite a wound completely, and may sometimes observe that at some places there is healing by first intention, while at others, after removal of the sutures, the wound gaps, and subsequently heals by suppuration." (p. 92.) It can hardly be questioned that the healing process is here one of *granulation*, and that suppuration is merely incidental.

The process of separation of dead from living sutesis is thus described:

"A cell infiltration and formation of vessels, leading to the development of granulations, start from the borders of the new tissue; granulations form on the border of the healthy tissue, and their surface breaks down into pus. With this change to the fluid state as it were the solution and melting of the tissue, of course the cohesion of the parts must cease, and the dead shreds, which previously were in continuity with the living tissue by their filamentary connection, must now fall."

We confess we can hardly conceive of the *solution* and *melting* of tissue. Living tissue does not thus *melt*, and whoever has tested the strength and tenacity of the tissue of a slough, must be convinced that it has successfully withstood *melting* influences. The process of amputating dead tissue, which nature institutes, is purely a vital one; to wit, *ulceration* or *destructive absorption of tissue*. It takes place in the living territory just adjoining the border of the dead. Granulation and suppuration are incidental. Granulation, *i. e.*, the building up of new tissue, certainly cannot effect the desired solution of continuity. And as organized tissue cannot be changed to pus, suppuration will be equally inefficacious. But tissue may be absorbed, and thus a solution of continuity effected. Such an absorption of *living* tissue just upon the borders of the dead territory effects the solution of continuity, by which a slough is cast off.

In *ulceration*, our author says:

"Two opposite processes are combined—new-formation and destruction; the latter results from liquefaction of the tissues, *i. e.*, through suppuration, or molecular disintegration, or both together." (p. 394.)

As *tissue* is not convertible into pus, suppuration cannot remove it; and molecular disintegration is *not* ulceration; it is merely minute mortification; in proof of which we quote our author, who says, in the same paragraph from which the last quotation is made, "Molecular disintegration and gangrene are but quantitative varieties of the same process, viz., the death of certain portions of tissue." Now the disconnection of these minute portions of dead tissue must be effected, and the process by which they are detached is the *real ulceration.* Examples of uncomplicated *ulceration*, *i. e.*, *destructive absorption of tissue*, are not wanting. Whoever has had opportunity for examination of diseased joints, must recall instances when a joint has been laid open which contained neither pus nor debris of any kind, but the cartilages of which were extensively *ulcerated*, *i. e.*, the cartilage tissue had disappeared in irregular patches; in other words, it had been the seat of *destructive absorption of tissue.*

In reference to the healing of nerves, Dr. Billroth says:

"In large superficial cicatrices, new nerves develop; when you have excised portions of skin and have brought together and united parts lying at a distance, new nerves grow through the cicatrix, and perfect power of conduction comes after a time, as may be often observed in plastic operations. These facts are very remarkable, and physiologically are still entirely inexplicable. Just think how wonderful that these nerve-filaments, sensory and motor, should find each other in the new adhesion, and that even, as we must suppose, the stumps of the primitive fibres should unite as they had been united, so that correct conduction and localization might result as they actually do!"

In the example cited there is only one kind of nerve function, viz., that of sensation; but in examples of reunion of divided compound nerve-trunks, which in inferior animals and, perhaps, in rare instances, in man, are attended by a complete restoration of lost power, no such accurate adjustment of the divided ends is probably necessary. It is not probable that there is more than one kind of nerve current or influence; and no histological difference has been discovered in nerve filaments, whether they minister to motion, sensation, or special sense. Prof. Allen taught in his lectures, twenty years since, and at a later date in a series of papers published in the *Medical Independent*, that the effect of the nervous current varied with the mechanism at the end of the nerve. If a nerve terminated in a muscle, motion was the result; if in the peculiar mechanism of a gland, secretion. If a nerve originated in the external skin, its current conveyed to the sensorium common sensation; if it started from an organ of special sense, special sensation. There has never been any proof of an *intrinsic* variety of nervous currents, and histology has not detected a variety of conducting nervous filaments.

Dr. Billroth gives a series of diagrams illustrating the succeeding phases in an organizing blood clot, with reference to which he says:

"It appears that in the clotted blood there is a cellular infiltration, (wandering cells) which here leads to development of connective tissue; in short, that the thrombus becomes organized. The thrombus is not a permanent tissue, but gradually disappears again, or, at least, is reduced to a minimum, a fate which it shares with many new formations resulting from inflammation."

Such an organization of coagulated blood is by no means improbable, and the observations of Dr. Billroth will tend to clear up this mooted point.

In the classification of tumors, the grouping is determined entirely by histology. Practically, we consider this arrangement of adventitious growths not only the least satisfactory, but really the most perplexing one that has yet been adopted. The natural history and clinical peculiarities of surgical diseases afford salient and recognizable features, which not only stamp varieties, but are also associated with prognosis and treatment. Histology is an element to be studied as a constituent of their pathology, but until histology becomes an end, and not a means, it will never serve a useful purpose as a basis of classification. Speaking of the course and progress of *sarcomata*, Dr. Billroth says:

"You see that the greatest benignity and greatest malignity may be united in this one group of neoplasia; indeed, I can assure you that two sarcomata of the most similar histological qualities (usually, however, with different consistence) may differ entirely in their course. From this circumstance the greatest objections have been made to pathological histology; it must be acknowledged that the histological structure of a tumor by no means corresponds to its clinical course; but for this reason to cast a slur on anatomy [histological anatomy, of course, he means] would be just as strange as to blame it because we cannot certainly distinguish between the microscopic preparations of a salivary, lachrymal, or mucous gland, although they play very different parts in the organism."

This would be very pertinent if anatomists made histology the basis of their description of the organs named. But histology is but a secondary matter to the descriptive anatomist. Classification serves the purpose of facilitating the description and study of tumors; to base classification on their histology, presents the subject to the student in as perplexing a form as would be the study of descriptive anatomy classified according to histological elements.

Billroth's Surgical Pathology will serve *us* a good purpose by using it as a reference in connection with Paget's; but we trust and believe that it will not soon supplant the latter work.

M. G.

*Elements of Medical Chemistry.* By B. Howard Rand, M.D., Professor of Chemistry in Jefferson Medical College. Second Edition, revised, with additions. Philadelphia: J. B. Lippincott & Co., 1871; pp. 420.

In this edition the text is thoroughly revised, an index added, as also a chapter on the clinical examination of the urine. We are pleased to see that the author has abstained from marring the general excellence of his convenient manual, by substitution of the proposed new nomenclature. He adheres to the nomenclature adopted in the U. S. Pharmacopœia. The constantly changing theories and hypotheses of dabblers in chemistry ought not to be allowed to upset a well established system, without better reasons than have yet been offered.

*Regimen Sanitatis Salernitanum.* Code of Health of the School of Salernum. Translated into English verse, with an Introduction, Notes and Appendix. By JOHN ORDRONAUX, LL.D., M.D., Professor of Medical Jurisprudence in the Law School of Columbia College, N. Y., etc., etc. Philadelphia: J. B. Lippincott & Co., 1871; pp. 167.

The original and translation are given on opposite pages, and it is unnecessary to say the work is well done. The author has laid the profession under obligations, by rendering this rare and interesting "code" accessible.

A limited edition (210 copies) was originally printed on extra large superfine tinted paper, for subscribers only. A few copies of that edition are still on hand, and can be obtained on application to the Publishers, at $5.00 a copy. We shall "scissor" some specimens when space permits.

*The Journal of the Gynæcological Society of Boston*: A monthly Journal, devoted to the advancement of the knowledge of the Diseases of Women. Edited by WINSLOW LEWIS, M.D., HORATIO R. STORER, M.D., GEORGE R. BIXBY, M.D. Vol. III, July to January, 1870. Boston: James Campbell, Publisher, 18 Tremont St., Museum Building; pp. 400.

We are gratified by a receipt of this volume elegantly bound in green and gold, for which the editors will please accept sincere thanks. "The Journal of the Gynæcological Society of Boston" is evidently thoroughly established, and has achieved the confidence of co-workers in the specialty to which it is devoted. In a literary way it wears the unmistakable marks of Bostonian "culture." Typographically, etc., it is unexceptionable, and even admirable. It is polemical to a degree—but that is an inherited peculiarity of its locality. Prof. Storer, who furnishes the principal motive power, is trenchant in attack, and potent in defence. He believes most fervently what he writes, and if we do not agree with him, in very many of his teachings, possibly the fault is our own.

The Society adopting as their motto, *Propter uterum est mulier*, religiously and logically carry out the implied dictum.

Those who desire to gain belief in that proposition, and acquaint themselves with all the modern methods of treating *the womb*, will find them expounded fully and forcibly in "The Journal of the Gynæcological Society of Boston," furnished to all advance paying subscribers at $5.00 per annum.

*The Causation, Course and Treatment of Reflex Insanity in Women.* By HORATIO ROBINSON STORER, M.D., LL.B., of Boston, Surgeon to St. Elizabeth's and St. Francis's Hospital for Women, etc., etc. *Sublata causa, tollitur effectus.* Boston: Lee & Shepard, Publishers. New York: Lee, Shepard & Dillingham, 1871; pp. 236.

This is a republication, in book form, of a paper which was communicated to the American Medical Association, at its session in Boston, in 1865, and was printed in its transactions for that year. It is republished at the present time, that it may appear simultaneously with a translation of a paper upon the relations of the female sexual organs, by Prof. Louis Mayer, of Berlin, which with notes by Prof. Storer, was commenced in the *Journal of the Gynæcological Society of Boston*, May, 1870. This essay, Dr. Storer remarks, "approaching the subject from a somewhat different standpoint, and in entire ignorance that he had been anticipated in the investigation by several years—affords unanswerable proof of the correctness of every point made in the following pages."

*Modern Therapeutics:* A Compendium of Recent Formulæ and Specific Therapeutical Directions. By GEO. H. NAPHEYS, A.M., M.D., one of the Editors of the Half Yearly Compendium of Medical Science, etc., etc. Second edition, revised and improved. Philadelphia: S. W. Butler, M.D., 115 South Seventh St., 1871; pp. 412.

The first edition of this work was noticed, at its appearance, in the *Journal.* It has been out of print for some months, all the issue having been sold in eight months. The author has performed the duty attempted in a very satisfactory manner, and has collated a mass of material not readily accessible to ordinary readers or busy practitioners. The facilities afforded by such books in every day practice, weighs largely in their favor against their admitted tendency to foster a formularized routine practice.

*A Hand Book of Legendary and Mythological Art.* By CLARA ERSKINE CLEMENT, Author of "A Simple Story of the Orient," with Descriptive Illustrations. New York: Published by Hurd & Houghton, Cambridge Riverside Press, 1871; pp. 497.

A capital book for the foreign traveler, or the home student. It brings together in compact shape all the principal Christian legends and Pagan myths, with concise expositions of their illustrations in the art galleries and museums of Europe. Numerous wood-cuts are given, and the subjects being discussed alphabetically, reference to any desired topic is made easy. It is just what is needed as a companion to the usual handbooks of European travel.

*American Association for the Cure of Inebriates.* Proceedings of the first meeting, held at New York, Nov. 29th and 30th, 1870. Philadelphia, 1871.

The perusal of this little pamphlet cannot fail to afford to every philanthropist the most sincere satisfaction, in the evidence, presented therein, of the attention which the subject of intemperance is just now attracting in the scientific world. Nor is it less satisfactory to perceive the tendency toward organization, in the efforts to eradicate this terrible scourge of humanity, or, at least, to counteract its influence.

As in the individual, considered in his physical relations, so in society there appears to be a principle of compensation, by means of which reactions from extremes are effected, and social adjustments elaborated out of the very elements which constituted the sources of disturbance. An evil, at first trivial, fails to attract attention, till, increasing in magnitude, it begins to disturb the essential equilibrium of society, and, in so doing, awakens the dormant instinct of self-preservation, and thereby effects its own suppression.

The efforts now made to effect the suppression of intemperance, seem to suggest the arrival of such an epoch in the progress of humanity, and while we by no means anticipate the speedy accomplishment of so desirable an end, nor indeed its accomplishment at all, by the means now in operation, we trust that this incipient reformation may itself undergo such continuous reformations as shall ultimately give it the form of truth and efficiency.

Believing most firmly in the maxim "*ne sutor ultra crepidam*," we feel, that, as medical observers, we are excluded from the consideration of the subject, in its moral, social and religious bearings, and that, while we have a most fruitful field of research in the elucidation of the terrible effects of intemperance upon the human organism, and a most useful, though gigantic labor in devising means for their prevention or removal, the suppression of the evil itself lies beyond our province, and properly belongs to religion, by which only it can be comprehended in all its phases, and by whose influence alone it can be successfully combatted and thoroughly eradicated.

Considered then from a medical standpoint, as the only point of observation appropriate for occupation by this Journal—the present phase of human society, at least in this country, presents many features peculiar to this epoch, and distinctly traceable to the influence of the various forms of intemperance; an influence which has undoubtedly induced in the human organism, considered as an individual, and in society as an aggregation of such individuals, modifications which, in their relations to the present and next succeeding generation, may be considered as permanent.

Much has been written about changes of type in disease, necessitating appropriate changes in its treatment; it is most reasonable to conclude that morbid influences, so far as they are specific and independent of causes external to themselves, have undergone no change at any time, but manifest varying symptoms through the medium of organizations which have sustained greater or less modifications through the operation of agencies among which intemperance must hold a most prominent place. As an illustration, the effects of intemperance, in inducing modifications of innervation, are prominently and painfully apparent to all who have devoted special attention to the pathology of this function; and in view of the constantly recurring instances of hereditary transmission of morbid susceptibility to alcoholism, their permanency (as above qualified) must unfortunately be admitted. Moreover, even where the direct relation of parental intemperance is not, as in the illustration just cited, so clear and definite, the multitudes of instances of insanity, of imbecility, and other manifestations of perverted innervation in the offspring of intemperate parents, indicate, if they do not demonstrate, the efficient agency of this cause in their production.

A very large share of the responsibility for the increased and increasing extent of intemperance is attributed to the medical profession, and while we, to a limited extent, assent to the justice of the opinion, we do not admit the reasons assigned therefor. While it is doubtless true that some, perhaps many, have commenced a career of intemperance by doses of alcohol prescribed by a physician, this incident has been, in the majority of cases, a mere pretext for the indulgence of an appetite already craving gratification, and certain to seek it otherwise had this means been wanting.

We think the responsibility of the medical profession lies rather in their misapprehension of the true physiological and therapeutic relations of alcohol; let these be clearly defined, as they certainly are not now, and while we may not perhaps hope, thereby, to reform confirmed inebriates, (for seclusion is not reformation), we might, by the formation of correct impressions upon minds yet untainted by the curse, anticipate and thus prevent their destruction.

But, craving pardon of our readers for this long digression, we return to "the address of the President" embodying four propositions, to which we venture to suggest only the following objections: We think the admission, in the reply to the second proposition, viz., that "in small doses it (alcohol) acts as a mild stimulant and tonic," is a fallacy, which has always constituted one of the strongest of the tippler's arguments, in defense of his practices, and which will continue to be thus abused until a correct appreciation of the physiological relations of alcohol shall demonstrate its fallacious character.

This theory that alcohol is a stimulant cannot be sustained upon correct physiological principles, and although it has received the endorsement of time, it is not less false than many other pseudoscientific opinions, which have passed current, under the sanction of the same authority, until overthrown by more logical conclusions from broader premises.

That, under the influence of moderate doses of alcohol, the consciousness of exhaustion of the normal proportions of vital force in any organ or system of organs, is diminished or suspended, is undoubtedly true, as demonstrated by the experience of the whole human race; and yet the process, by which sensibility, whether mental or physical, is lessened, cannot properly be termed stimulation.

Nor is this theory less mischievous than fallacious. The human intelligence associates with the expression stimulation, the idea of increased power, and becoming conscious of loss of power in any function, naturally seeks some means for its restoration, and reasonably looks to those agents classified as stimulants for the supply of such means.

There are but two agents out of which force can be elaborated in the organism, viz., food, and sleep (*i. e.*, rest). Alcohol is not food, hence in this relation of the laws of force, it has little value. Impress the mind of man with the conviction, that alcohol can never in any sense increase power, but can only by lowering the energy (force in action) of the sensory function, the true indicator of disturbance of force, diminish sensibility to the loss of power already sustained, and thus add to the pre-existing depression—accomplish this, and a new and true principle will be established upon which to base a broader system of reform.

To the fourth proposition, setting forth that "the object of this meeting is to inquire into the best mode of treating inebriety," we object, that the second subdivision implicitly defines the investigation, and virtually terminates further inquiry.

Appreciating, at their highest value, the good intentions of the Association, whose organization is herein announced, it is with much regret that we see the reform movement taking its origin in principles which being narrow, partial, and fallacious, can only serve as foundations for delusive theories, whose practical application must result in disappointment.

The importance of the work to which the Association proposes to devote itself, the prominent position which it already occupies in the public view, and the eminence of some, at least, of its members, would, we think, justify a careful revision of its "Declaration of Principles;" for we cannot believe that to be a "true method for the recovery of inebriates," which is based upon the assumption that "intemperance is a disease"—an assumption which no pathologist can prove, and every lexicographer contradicts.

Further, that, "its primary cause is a constitutional susceptibility to the alcoholic impression" we doubt, for we could designate, by name, individuals possessed of the most intense susceptibility to the influence of alcohol, who have always been models of temperance, and to whom this very susceptibility has proven a protection and a safe-guard against this "disease," rather than, as here asserted, "its primary cause."

To those uninitiated in the mysteries of the law, legal fictions have ever been a source of "wonder and doubt," but comprehensive as their scope may be, we trust that it will never be enlarged by the assertion of the principle embodied in the eighth proposition, viz., "That the law should recognize intemperance as a disease." The law is certainly in this instance wiser than the philanthropists, in designating intemperance as a vice, whatever may be the nature of its causes or results.

The writer of a "Brief paper on the pathological influences of alcohol, and the nature of inebriation," might have remembered the Horatian maxim, "*brevis esse laboro, obscurus fio*," with the advantage of clearing some of its obscurities; for after the fifteenth reading of the first paragraph of the fifteenth page, we are still in doubt, whether he endorses "fermented and distilled drinks" as "tonic, nutritive and life sustaining" when "properly used," or denounces the rest of mankind for doing so—more especially when, upon the next page, he refers to the "direct exhilarating effect of alcohol on the nervous system."

It would also have been interesting, had he explained on the twenty-second page, how alcohol "modifies the play of vital affinity in such a manner as to retard atomic and molecular changes," etc.

Another essayist places inebriates in the class described by Maudsley, as "weighted with a destiny, against which they have neither the will nor the power to contend"—with "primarily defective condition of body and mind, and an impaired will"—

who "know, but are impotent to perform," etc. All of which conditions we are led to believe are to be removed by temporary seclusion in an asylum. *Credat Judæus Apella, non ego.*

The Reverend author of the essay on the "Relation of the church to inebriates," states some plain truths, when he asserts that "a large proportion of our inebriates first began to drink, because their religious training had been overlooked, and there was no fear of God before their eyes from their youth up"—that "the church is letting go its hold upon the horny-handed sons and daughters of toil," having "no place provided where the victims of sin and shame, the ragged and battered and torn, can stand or bow down to worship God," and the "utter failure of the church to carry out the principles of pure religion and undefiled"—"to visit the fatherless and the widows in their affliction." In all of which we most heartily agree, more especially as he restricts his meaning to his own church, of which, speaking *ex cathedra*, he speaks only the truth.

Finally, in expressing our warmest sympathy with the Association in its legitimate career, we will venture to express the hope that it will temper zeal with discretion, and in its efforts for the reform of inebriates, will not exclude from the folds of its mantle of charity, those who are so unfortunate as to be sober and temperate, and above all things let it withhold its reforming hands from the law, and refrain from swelling the mass of special legislation now encumbering every statute-book—destined to prove unfailing sources of confusion and corruption. W. H.

### PAMPHLETS RECEIVED.

*The Ophthalmoscope in the Treatment of Epilepsy.* By Reuben A. Vance, M.D., Attending Physician for Diseases of the Nervous System at the Out-door Department of Bellevue Hospital, etc., etc. New York: D. Appleton & Co., 1871.

*Vick's Illustrated Catalogue and Floral Guide.* 1871. James Vick, Rochester, N. Y.; pp. 96. Profusely illustrated.

*Annual Report of the Board of Health to the General Assembly of Louisiana.* Dec. 31st, 1870; session 1871. New Orleans: John W. Madden, printer, 73 Camp street; pp. 83.

*On Dactylitis Syphilitica, with Observations on Syphilitic Lesions of the Joints.* By R. W. Taylor, M.D., Surgeon to the New York Dispensary, Department of Venereal and Skin Diseases. New York: F. W. Christern, publisher, 1871; pp. 30.

*Report on Otology.* By Samuel J. Jones, A.M., M.D., Lecturer on Ophthalmology and Otology, etc., etc. Read before the Twentieth Anniversary Meeting of the Illinois State Med. Soc.; pp. 14.

*Woman as a Physician.* By J. P. Chesney, M.D., New Market, Missouri. From the author; pp. 15.

*Health and Home.* A Monthly Magazine Devoted to Health and the Home Circle. W. R. DePuy & Brother, publishers, 805 Broadway, New York; $1.50 per annum; Vol. 1, No. 1; pp. 32.

*The Health and Wealth of the City of Wheeling; also General Remarks on the Natural Resources of West Virginia.* By James E. Reeves, M.D., City Health Officer, and Author of a Practical Treatise on Enteric or Typhoid Fever. Second edition, enlarged and illustrated; price 60c.; pp. 158.

*Archives of Science and Transactions of the Orleans Co. Soc. of Natural Science.* Jan. 1871. Published quarterly by J. M. Crurier, M.D., Newport, Orleans Co., Vermont.

*The Georgia Medical Companion.* A Monthly Adviser, devoted to the Science of Medicine and Surgery. Edited by T. S. Powell, M.D., and W. T. Goldsmith, M.D. *Quicquid Præcipies Esto Brevis.* Atlanta, Georgia: J. J. Toon, publisher; $2 a year.

